# Peak external game demands are impacted by season phase but not match outcome or a mid-season coaching change in semi-professional, male basketball players

**DOI:** 10.3389/fphys.2026.1778157

**Published:** 2026-04-22

**Authors:** Abel Ruiz-Álvarez, Enrique Alonso-Pérez-Chao, Álvaro Bustamante-Sánchez, Aaron T. Scanlan, Miguel-Angel Gómez-Ruano

**Affiliations:** 1 Facultad de Ciencias de la Actividad Física y del Deporte, Universidad Politécnica de Madrid, Madrid, Spain; 2 Facultad de Ciencias de la Vida y la Naturaleza, Universidad de Nebrija, La Berzosa, Madrid, Spain; 3 Faculty of Medicine, Health and Sports, Department of Sports Sciences, Universidad Europea de Madrid, Madrid, Spain; 4 Department of Physical Activity and Sports Science, University Alfonso X el Sabio, Madrid, Spain; 5 Department of Sports, Exercise and Health Sciences, University of Trás-os-Montes and Alto Douro, Vila Real, Portugal; 6 Research Center in Sport, Health and Human Development (CIDESD), Vila Real, Portugal; 7 School of Health, Medical, and Applied Sciences, Central Queensland University, Rockhampton, QLD, Australia; 8 S.P.O.R.T. Research Cluster, Central Queensland University, Rockhampton, QLD, Australia

**Keywords:** contextual factors, high-intensity periods, monitoring, player load, training periodization

## Abstract

**Introduction:**

Basketball competition imposes high physical demands on players. Quantifying the peak demands (PD) encountered is useful to identify the most intense passages of play, with more research needed to better understand factors that may impact PD. Therefore, this study aimed to examine the effects of season phase, match outcome, and a mid-season coaching change on PD in semi-professional, male basketball players.

**Methods:**

Eleven players from a single team competing in the Spanish fourth division were monitored across 33 official matches during the 2021–2022 season. PlayerLoad™ (PL) was measured using a validated local positioning system and analyzed across 30-s, 60-s, and 180-s rolling windows. Contextual variables included season phase (preseason, first half, second half, and promotion), match outcome (win and loss), and coaching change (before and after a mid-season change in head coach). Friedman and Wilcoxon tests were used to compare PD between conditions.

**Results:**

Season phase significantly (p <0.05) influenced PD across all time windows (30 s, 60 s, and 180 s), with higher values observed during the pre-season and promotion phases compared to both halves of the regular season. PD over 180 s was also higher in the pre-season than in the promotion phase. Regarding match outcome, PD over 30 s was significantly greater in losses than in wins (p = 0.0049, large effect), while 60 s and 180 s windows showed non-significant, large trends in the same direction. No significant differences were found in PD before versus after the mid-season coaching change, suggesting no meaningful impact on these external demands.

**Conclusion:**

These findings may help inform player training prescriptions and preparatory plans aligning with fluctuations in match demands across season phases.

## Introduction

Basketball is a multifaceted team sport requiring players to perform repeated high-intensity efforts, including accelerations, decelerations, jumps, and changes of direction, while simultaneously executing complex technical and tactical decisions in time-constrained and high-pressure environments (Stojanović et al., 2018). The ability to sustain such efforts consistently throughout a game, recover quickly between actions, and maintain performance under fatigue is essential for competitive success (Antoranz et al., 2024; Espasa-Labrador et al., 2023). Given these demands, training design must align with match-play to optimally prepare players to withstand competitive stimuli (Sosa et al., 2025). In this regard, understanding the physical intensities required of players during matches is of particular interest for practitioners. The two principal approaches adopted to assess the physical intensities encountered in basketball match-play include quantifying the: (i) average demands across an entire match; and (ii) peak demands (PD) that represent the most intense passages within rolling time windows (e.g., 30 s, 60 s, 180 s) (Alonso et al., 2020; García et al., 2021; Vázquez-Guerrero and Garcia, 2021).

Quantifying the average intensity encountered across entire matches disregards the stochastic nature of match activities by smoothing out critical spikes in physical demands that can occur (Alonso et al., 2020). Consequently, relying on average intensity data to inform player preparation may potentially leave them unprepared for the most demanding passages encountered in competition (Gabbett, 2020; Irid et al., 2025; Pérez-Chao et al., 2023a). Alternatively, quantifying the PD across specific durations using rolling average analyses has emerged as a more precise approach to understand the intensities that are reached throughout match-play (Antoranz et al., 2025; Cunningham et al., 2018; Pérez-Chao et al., 2023a). Recent evidence reinforces the unique insight afforded by quantifying PD, which can exceed average values determined throughout entire basketball matches by ~3–7 times across different external load variables (Vázquez-Guerrero et al., 2020). Consequently, the integration of PD-based metrics into conditioning programs may better prepare players, who are capable of sustaining high intensities for requisite durations under fatigue and competitive pressure (Gabbett et al., 2019).

Several contextual factors may impact the external loads, including the PD, encountered during basketball competition (Pérez-Chao et al., 2023a; Sansone et al., 2025). Among these contextual factors, season phase may play a decisive role in modulating training and match demands. For instance, preseason periods typically involve training approaches that emphasize general fitness and load tolerance, with matches used to prepare players for the upcoming competitive season (Buchheit and Laursen, 2021; Haff, 2021). In turn, training plans during the regular season must strike a balance between recovery, physical development, and tactical refinement to optimize player readiness for regular competition in alignment with the schedule faced (Buchheit and Laursen, 2021; Mujika et al., 2018; Robertson and Joyce, 2018). However, fluctuations in the PD across seasonal phases remain poorly understood in the basketball literature, with no structured analyses comparing between distinct periods throughout the full season. For instance, descriptive analyses showed that the proportion of time spent in games and training, as well as completing different drills in training contexts, varied between the pre-season and regular season phases among professional players from a National Basketball Association team (Russell et al., 2021b). Moreover, rudimentary analyses revealed comparable external PD (30-s, 60-s, and 180-s windows) between friendly matches–as typically performed in the pre-season phase–and competitive matches during the regular season among professional and semi-professional, male basketball players (Antoranz et al., 2025). Insight into how PDs fluctuate throughout the season may assist basketball practitioners in optimizing training loads to better prepare players for competition and mitigate their injury risk.

Further contextual factors that have received limited attention in the basketball literature include the impact of match outcome and different head coaches for the same team on the PD experienced during matches. Regarding match outcome, it has been posited that teams may exert more intense physical outputs to build leads in achieving a winning outcome, or alternatively to reduce deficits in the match scoreline when in a losing position (Fox et al., 2021). However, the small body of research exploring this factor has largely observed non-significant differences in PD (using several load variables) between matches there won and lost, including in under-18 years, regional, male players (Pérez-Chao et al., 2023b), national-level, male players (Antoranz et al., 2024), and semi-professional, male players (Fox et al., 2021). Nevertheless, further research is necessary to confirm whether these initial findings regarding the negligible differences in PD based on match outcome remain consistent in wider basketball player samples than the few previously investigated. Regarding coaching changes, no research has explored the impact of this factor on PD in any basketball setting to date. Related research has shown that variability in external load periodization exists across professional basketball coaches regarding the daily loads (Salazar et al., 2024) and weekly loads (Salazar et al., 2020) administered. Consequently, such variation in load management plans, combined with potential differences in tactical strategies across coaches, may theoretically translate to variations in the PD encountered during match-play under different coaches. In particular, coaching philosophy may influence key game characteristics such as tempo, defensive schemes, transition frequency, and substitution patterns, which can modify the frequency and intensity of the most demanding passages of play (Castillo et al., 2021). However, investigating this factor is difficult given coaching changes often occur between seasons, making it difficult to discern the impact of other confounding factors (e.g., player turnover, match scheduling) on PD in such scenarios. In turn, coaching changes made during the season present a useful opportunity to more directly explore the impact of the head coach on the PD encountered during match-play within the same players.

Consequently, this exploratory study aimed to examine the impact of contextual factors–season phase, match outcome, and mid-season coaching change–on PD in semi-professional, male basketball players. Understanding how these variables affect PD may help inform the development of more individualized and context-sensitive training and preparation strategies throughout the season.

## Materials and methods

### Samples

Male basketball players (n = 11, age: 21.3 ± 5.3, height: 196.5 ± 6.8 cm, body mass: 88.53 ± 10.75 kg) from the same team competing in the fourth national Spanish basketball division (Spanish Amateur Basketball League), with a competition frequency of one official game per week, were monitored throughout the 2021–2022 season (October to April). Across the 33 matches analysed, a total of 315 individual player observations were obtained from the 11 players who met the inclusion criteria. However, for the statistical analyses, repeated observations within each player were collapsed into a single representative value (median) per condition to avoid pseudo-replication. Consequently, inferential analyses were conducted at the player level, whereby a total of 11 players (positions defined by the head coach as guards: n = 3; forwards: n = 3; and centers: n = 5) participated in this study. Players typically completed four on-court team training sessions per week, as well as strength, conditioning, and individual technique sessions. The inclusion criteria for this study involved players needing to have participated for a minimum of 15 min of playing time per match across at least one third of the total matches completed (i.e., a minimum of 11 out of 33 matches). While no standard has been established regarding inclusion criteria of this nature for monitoring studies in basketball, this approach was adopted to ensure a robust dataset was obtained where players were able to consistently register PD across all epochs of interest throughout different season phases. Players were informed of the purpose, risks, and benefits of participation in the study before providing written consent (or written assent alongside guardian consent if under 18 years of age) in alignment with the Declaration of Helsinki (Hellmann et al., 2014). All player data were anonymized, with confidentiality maintained throughout the study.

### Procedures

This exploratory study employed a retrospective, longitudinal, observational design. Data were collected throughout an entire season and encompassed 33 official matches involving semi-professional, male basketball players. Throughout matches, each player was equipped with a monitoring device (Vector S7, Catapult Sports, Melbourne, Australia), which was placed in a fitted neoprene vest worn underneath standard playing attire. The device was positioned over the upper thoracic spine, between the scapulae (Hodder et al., 2020). Each device contained an accelerometer ( ± 16 g, 100 Hz), magnetometer ( ± 4.900 µT, 100 Hz), and gyroscope (up to 2,000 deg/sec, 100 Hz). All players were familiar with monitoring technology as they had worn the devices during training sessions and matches in the previous season. Devices were turned on ~20–40 min before the warm-up before each match, and players wore the same device throughout the study period to avoid inter-unit variation in outputs (Castellano et al., 2011).

### Variables

PD were calculated using PlayerLoad™ (PL) values expressed in absolute terms and extracted for each player across three rolling time windows, including 30 s, 60 s, and 180 s. These epochs were chosen, given they are among the most commonly used in basketball research and have strong practical application in basketball teams (Pérez-Chao et al., 2023a). All data were obtained directly from the Catapult OpenField software (version 3.11.0) accompanying the monitoring devices, which enables automated computation of PD for predefined time intervals. Peak values were automatically extracted using the integrated rolling average method for detecting PD. This method continuously analyzes performance data over moving time intervals to identify the period eliciting the highest intensity for each epoch (Cunningham et al., 2018). Once extracted, all PD data were organized into customized Microsoft Excel spreadsheets (version 16.0, Microsoft Corporation, Redmond, WA, USA) for further analysis.

The impact of three key contextual factors on PDs were explored in this study – season phase, match outcome, and mid-season coaching change. Firstly, the season phase was categorized into four distinct periods to capture temporal changes in PD across the season. The first period was the pre-season phase (3 matches – 20 individual data samples), spanning from the initial preparation period up to the start of official competition and including early friendly matches (August–September). The second period was the first half of the regular season (16 matches – 160 individual data samples), spanning from the first match in the regular season until each opponent had been played once (September–January). The third period was the second half of the regular season (12 matches – 115 individual data samples), spanning from the end of the first half of the regular season until the commencement of the promotion stage (January–April). The fourth period was the promotion stage (2 matches – 20 individual data samples), which involved competition against high-level opponents to secure promotion to a higher division (April–May). Secondly, match outcome was categorized as a win (26 matches – 251 individual data samples) or a loss (7 matches – 64 individual data samples) based on the final result of each match. Thirdly, coaching change was categorized into matches completed under the head coach who started the season in August and concluded their tenure in January (18 matches – 170 individual data samples) and matches completed under the head coach, who was externally appointed mid-season (January) and continued for the remainder of the season (15 matches – 145 individual data samples). Overall, 315 samples were included in the analysis among the 11 players.

### Statistical analysis

Due to violations of normality assumptions across most variables detected via the Shapiro-Wilk test, non-parametric analyses were conducted. Each player produced multiple PD observations within each contextual condition across the season. To avoid pseudo-replication arising from repeated observations within players, these observations were collapsed into a single representative value per player for each condition by calculating the median. The median was selected as a robust measure of central tendency given the presence of non-normal distributions and potential extreme values in peak demand data. Consequently, each player contributed one datapoint per condition for all contextual comparisons (n = 11). Analyses were therefore conducted using paired data at the player level and descriptively reported as medians and interquartile ranges at the group level. Differences in PD across season phases (pre-season, first half of the regular season, second half of the regular season, and promotion phase—i.e., the playoff stage determining promotion to a higher division) were examined using Friedman tests. Where significant effects were observed, *post hoc* comparisons were conducted using Wilcoxon signed-rank tests with Bonferroni-adjusted significance levels. Effect sizes for the Friedman tests were assessed using Kendall’s W and interpreted as trivial (<0.10), small (0.10–0.29), moderate (0.30–0.49), or large (≥0.50). Differences in PD according to match outcome (wins vs losses) and mid-season coaching change (before vs after coaching change) were examined using Wilcoxon signed-rank tests. Effect sizes were calculated using rank-biserial correlations (r_rb_) and interpreted as trivial (≤0.10), small (0.11–0.30), moderate (0.31–0.50), or large (>0.50) (Rea and Parker, 2014). Statistical significance was set at p <0.05. The IBM SPSS statistical package for Windows (version 24.0, IBM Corp., Armonk, NY) and the Jamovi statistical software for Windows (version 2.5, Jamovi Project, Sydney, Australia) were used to analyse data.

## Results

**Figure 1:**
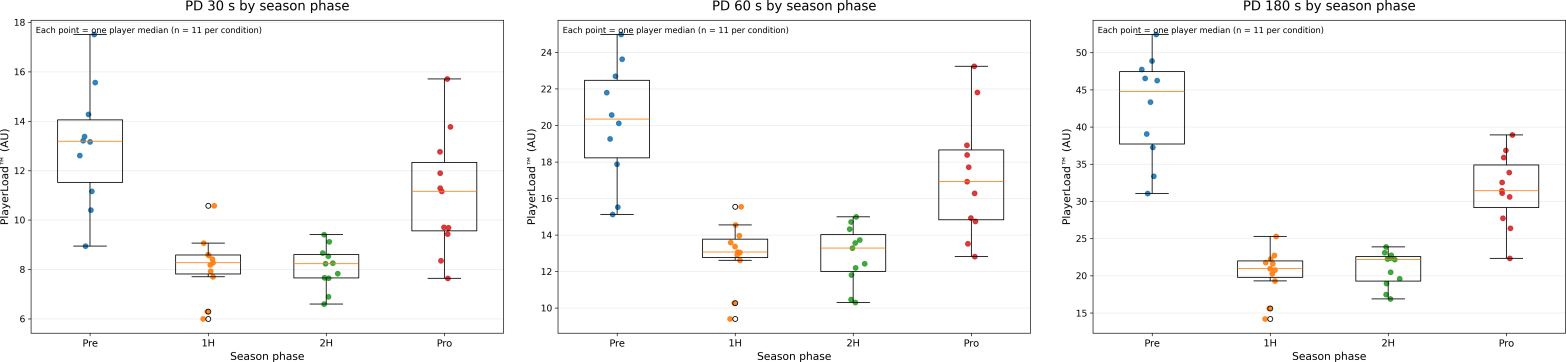
Box plots displaying the distribution of peak demands (PD) data according to the season phase. Each data point represents the aggregated value for an individual player (median across matches within each phase; n = 11 per condition). Box plots represent the group median and interquartile range based on these player-level values, with whiskers extending to the most extreme values within 1.5 times the interquartile range. PD was quantified using PlayerLoad™ (arbitrary units).

**Table 1** shows the descriptive PD data and comparison statistics for season phase analyses. For further visual interpretation, **Figure 1** displays box plots representing the distribution, density, and central tendency of the PD data according to season phase. Friedman analyses revealed a significant main effect of season phase on PD across all time windows (30 s: χ²(3) = 24.48, p <0.001, W = 0.816, large effect; 60 s: χ²(3) = 25.92, p <0.001, W = 0.864, large effect; 180 s: χ²(3) = 26.40, p <0.001, W = 0.880, large effect). *Post hoc* Wilcoxon signed-rank tests indicated that PD were significantly higher in the pre-season than in both halves of the regular season across all time windows (r_rb_ = 1.00, large effects). Similarly, PD during the promotion phase significantly exceeded those observed in the first and second halves of the regular season (r_rb_ = 1.00, large effects), and PD over 180 s were significantly higher in the pre-season than in the promotion phase (r_rb_ = 0.80, large effect).

**Table 1:** Descriptive and comparison statistics for peak demands (PD) according to season phase in semi-professional, male basketball players.

Variable	Season phase				Comparison statistics			
	*Pre-season*	*First half*	*Second half*	*Promotion*	*χ² (3)*	*p*	*Kendall’s W*	*Post-hoc* *(p <0.05)*
PD 30 s	12.9 (1.6)	8.4 (1.9)	8.2 (1.5)	10.6 (3.8)	24.48	<0.001	0.816 (large effect)	Pre > 1H, 2H (r_rb_=1.00) Pro > 1H, 2H (r_rb_=1.00)
PD 60 s	20.2 (3.4)	13.6 (3.0)	13.4 (2.7)	16.1 (5.9)	25.92	<0.001	0.864 (large effect)	Pre > 1H, 2H (r_rb_=1.00) Pro > 1H, 2H (r_rb_=1.00)
PD 180 s	42.1 (8.5)	21.5 (5.3)	22.1 (4.2)	29.4 (14.7)	26.40	<0.001	0.880 (large effect)	Pre > 1H (r_rb_=1.00), 2H (r_rb_=1.00), Pro (r_rb_=0.80) Pro > 1H (r_rb_=1.00), 2H (r_rb_=1.00)

Values are reported as medians (interquartile ranges) derived from individual player data. Repeated observations across matches were collapsed into a single representative value per player within each condition (n = 11). Differences between season phases were assessed using a Friedman test, followed by Wilcoxon signed-rank *post hoc* comparisons with Bonferroni-adjusted significance levels. PD was quantified using PlayerLoad™ (arbitrary units). Effect sizes for the Wilcoxon *post hoc* comparisons are reported as rank-biserial correlations (r_rb_) and interpreted as trivial (≤0.10), small (0.11–0.30), moderate (0.31–0.50), or large (>0.50).

**Figure 2:**
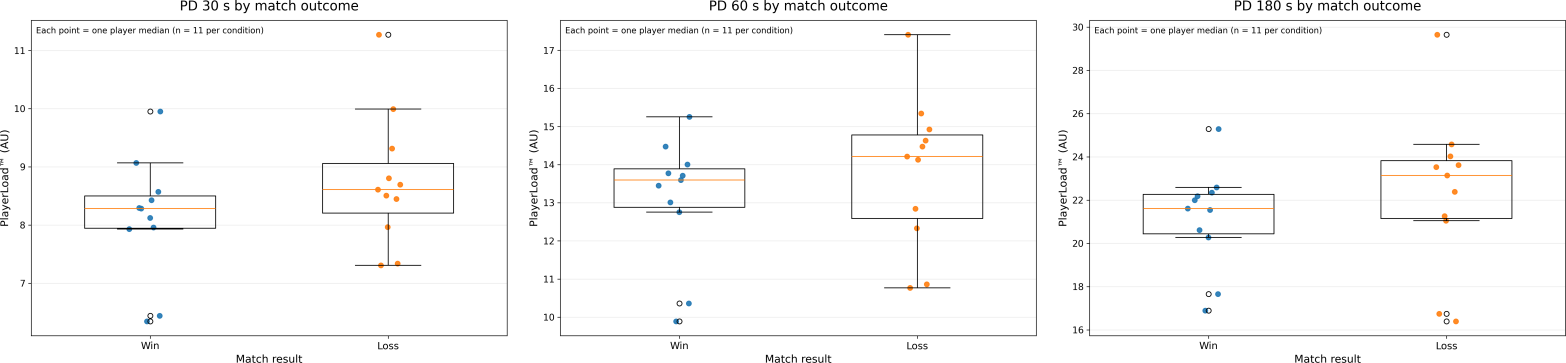
Box plots displaying the distribution of peak demands (PD) data according to match outcome. Each data point represents the aggregated value for an individual player (median across matches within each condition; n = 11 per condition). Box plots represent the group median and interquartile range based on these player-level values, with whiskers extending to the most extreme values within 1.5 times the interquartile range.

**Table 2** presents the descriptive PD data and corresponding comparison statistics according to match outcome. For enhanced visual interpretation, **Figure 2** displays box plots illustrating the distribution of PD across time windows. When comparing match outcomes using paired analyses, PD over 30 s was significantly higher in matches that were lost compared with those that were won (*p* = 0.005, r_rb_ = 0.91, large effect; W = 0, *n* = 11). The Wilcoxon statistic (W = 0) indicates that the differences between conditions were highly consistent across players, with all paired observations showing the same directional pattern. Although median differences appeared relatively small, the large rank-biserial correlation reflects a consistent separation between the distributions, indicating that values from one condition were systematically higher than those from the other. PDs over 60s and 180s showed non-significant trends toward higher losses, with large effect sizes.

**Table 2:** Descriptive and comparison statistics for peak demands (PD) according to match outcome in semi-professional, male basketball players.

Variable	Match outcome		Comparison statistics		
	*Win*	*Loss*	*W*	*p*	*r_rb_*
PD 30 s	8.41 (1.88)	8.92 (2.11)	0	0.005	0.909 (large)
PD 60 s	13.6 (3.2)	14.1 (3.5)	12	0.054	0.667 (large)
PD 180 s	21.7 (6.2)	22.4 (6.0)	14	0.667	0.636 (large)

The match outcome was categorized as a win or a loss based on the final result. Values are reported as medians (interquartile ranges) derived from individual player data. Repeated observations across matches were collapsed into a single representative value per player within each condition (n = 11). Differences between match outcomes were assessed using Wilcoxon signed-rank tests. PD was quantified using PlayerLoad™ (arbitrary units). Effect sizes are reported as rank-biserial correlations (r_rb_) and interpreted as trivial (≤0.10), small (0.11–0.30), moderate (0.31–0.50), or large (>0.50).

**Figure 3:**
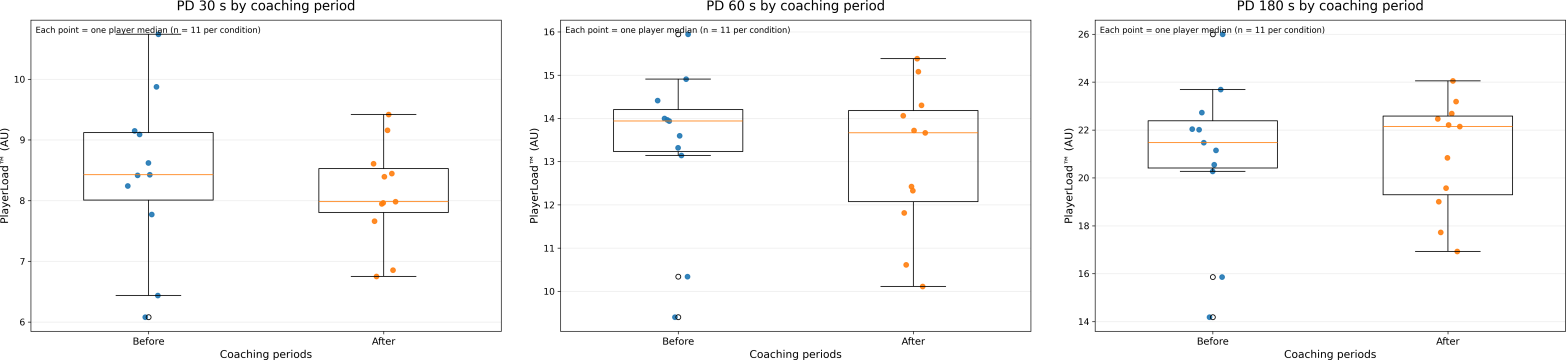
Box plots displaying the distribution of peak demands (PD) data according to mid-season coaching change. Each data point represents the aggregated value for an individual player (median across matches within each condition; n = 11 per condition). Box plots represent the group median and interquartile range based on these player-level values, with whiskers extending to the most extreme values within 1.5 times the interquartile range. PD was quantified using PlayerLoad™ (arbitrary units).

**Table 3** presents the descriptive PD data and comparison statistics for analyses according to mid-season coaching change, with further visual interpretation of these data provided in **Figure 3** via box plots. No significant differences in PD were observed before versus after the mid-season coaching change for any time window (all p >0.0, trivial-to-small effects), indicating that the coaching change did not meaningfully influence peak external demands during matches.

**Table 3:** Descriptive and comparison statistics for peak demands (PD) according to mid-season coaching change in semi-professional, male basketball players.

Variable	Mid-season coaching change		Comparison statistics		
	*Before*	*After*	*W*	*p*	*r_rb_*
PD 30 s	9.52 (1.42)	9.08 (1.36)	24	0.413	−0.303 (moderate)
PD 60 s	15.2 (1.51)	14.8 (1.96)	26	0.520	−0.242 (small)
PD180 s	25.8 (3.62)	25.1 (3.58)	30	0.831	−0.091 (small)

Categories are presented as “before” and “after” the mid-season coaching change to represent periods under each head coach. Values are reported as medians (interquartile ranges) derived from individual player data. Repeated observations across matches were collapsed into a single representative value per player within each condition (n = 11). Differences between conditions were assessed using Wilcoxon signed-rank tests. PD was quantified using PlayerLoad™ (arbitrary units). Effect sizes are reported as rank-biserial correlations (rrb) and interpreted as trivial (≤0.10), small (0.11–0.30), moderate (0.31–0.50), or large (>0.50).

## Discussion

The primary aim of this study was to examine the impact of contextual factors (season phase, match outcome, and mid-season coaching change) on PD in semi-professional, male basketball across an entire season. The main findings revealed that season phase significantly impacted PD, which were higher during the pre-season phase and the promotion stage than the first and second halves of the regular season. These findings may be explained by the unique contextual characteristics of the pre-season phase and the promotion stage. More precisely, elevated external PD in the pre-season may stem from the absence of refined tactical organization and structured match-play. Without established playing systems, play tends to be more disorganized and chaotic, leading to more frequent transitions, turnovers, and unstructured movements, all of which may increase the physical intensity across brief periods. Additionally, pre-season matches may present greater variability in opponent quality, increased experimentation with player rotations, lower tactical cohesion between teams, and heterogeneous levels of competitiveness, which may further contribute to elevated physical demands; however, the most recent reviews have not specifically examined or systematically analysed the influence of these factors (Pérez-Chao et al., 2023b; Sansone et al., 2025). As teams mature technically and tactically, they develop a more deliberate rhythm of play that defines their strategic identity.

Conversely, in the promotion stage, higher PD may stem from the superior competitive level of opponents and the critical nature of these matches. The intensity of play likely increases given the rise in stakes regarding match outcomes in the promotion stage (i.e., promotion to a higher division). Similarly, Palmer et al. (2022) observed greater training intensities and higher levels of vigorous activity during finals compared to the regular season in semi-professional, male and female basketball players. Starters also played more minutes, performed more maximal activity, and spent less time inactive during finals in this previous study. These findings align with our results, suggesting that as competition importance increases, players are required to execute more intense physical efforts sustained across epochs of varying durations to meet the tactical and situational demands of decisive matches. Taken together, the combination of tactical disorganization in pre-season and elevated opposition within high-stakes settings during the promotion stage may explain why these phases elicit higher PD. These findings underscore the importance of interpreting physical intensities within the appropriate competitive context, particularly with respect to variations across season phases.

In contrast, match outcome and a mid-season coaching change did not significantly impact the PD encountered during matches. These findings add to the limited research on this topic that aligns with our results, reporting that PD measured across varying rolling time windows do not significantly differ between matches that were won and lost. More precisely, Pérez-Chao et al. (2023b) reported non-significant, trivial–small differences in PD determined across different epochs (30 s, 60 s, and 300 s) with various external load variables (PlayerLoad™, accelerations, decelerations, and distance covered overall and at varying speeds) between matches that were won (8 matches) and lost (1 match) in under-18 years, regional, male players. Similarly, Antoranz et al. (2024) observed non-significant, trivial differences in PD determined via PlayerLoad™ across 30-s, 60-s, and 180-s time windows between matches that were won (21 matches) and lost (8 matches) in national-level, male players. In turn, Fox et al. (2021) analyzed the impact of outcomes in individual quarters (i.e., match quarters that were won vs. those that were lost), also reporting non-significant, trivial–small differences in PD determined via PlayerLoad™ across various time windows (15 s, 30 s, 60 s, 120 s, 180 s, 240 s, and 300 s) in semi-professional, male players. While research is still emerging in this area, the growing collective evidence emphasizes that PD determined across various epochs with different external load variables are consistent in winning and losing scenarios across various male basketball player samples. In this regard, simply maximizing the intensities performed across brief periods might not necessarily translate to winning outcomes, with team performance likely much more complex and predicated on the interactions among many elements (e.g., physical, physiological, technical-tactical, psychological, environmental, and sociological factors) (Rogers et al., 2022; West et al., 2021). Consistent with this notion, previous studies have reported weak or non-significant associations between external load metrics and match performance indicators in basketball (Fox et al., 2022; García et al., 2022), implying that greater physical outputs do not inherently result in superior performance.

Regarding the mid-season coaching change, no significant differences in PD were observed across any time window, with trivial-to-small effect sizes. These findings suggest that the coaching transition did not meaningfully influence PD during official matches. This is the first study to examine the impact of a mid-season coaching change as a contextual factor on PD in basketball. As highlighted previously, elite coaches at the EuroLeague level exhibit distinct prescriptive differences in the daily external loads their players encounter surrounding matches (Salazar et al., 2024) and across the week (Salazar et al., 2020). However, these data are reflective of coaches from different teams in the same season, coaches across different seasons, coaches with different player rosters within the same team, and mostly training settings. Each match presents a unique tactical challenge, requiring the coaching staff and players to adapt their strategic preparation based on the opponent’s profile. This consideration includes anticipating how to manipulate game rhythm and structure to gain a competitive advantage. Variations in pace, playing times accumulated, and fatigue-related variables may subsequently influence the physical demands encountered during play (Alonso Pérez-Chao et al., 2022, 2021; García et al., 2020). Nonetheless, our findings did not reveal meaningful differences in PD attributable to the mid-season coaching change. This consistency likely reflects the limited opportunity for substantial tactical modification when a change occurs in mid-season, as teams generally maintain their established systems and style of play. In such cases, coaching changes may be driven more by psychological or motivational factors, such as enhancing team morale or responding to underperformance, rather than by intentions to alter external loading. While subtle tactical adjustments may arise between coaching periods, our data indicates that these do not translate into meaningful changes in the PD imposed on players. As player tracking technologies continue to advance, future research may better clarify the extent to which a coach’s tactical imprint modulates the physical demands in competition.

While this study provides novel insights into how contextual factors impact PD in semi-professional, male basketball players, several limitations must be acknowledged when interpreting the presented findings. First, the analysis focused exclusively on PL as the primary external load metric, which, although widely used in basketball research (Russell et al., 2021a), it encompasses holistic movement demands without isolating loading for key movements integral to basketball performance like accelerations and jumps. Second, when segmenting the sample by match result or season phase, there was a substantial imbalance in the number of samples per category – which were particularly low for the pre-season phase and promotion stage and for matches that were lost given the reduced sampling opportunities in these categories. Moreover, the recruitment of a single team across one season prohibited analyses considering how the examined contextual factors interact together (e.g., matches won or lost within a specific season phase). This scenario is commonplace in exploratory, observational research in applied sport environments but potentially limits the robustness of some findings. Future longitudinal studies spanning multiple seasons and teams may overcome these issues to help reinforce the observed outcomes. Third, the tactical and strategic approaches of the coaching staff before and after the mid-season change were not monitored, making it unclear whether such aspects were modified. In this regard, although no meaningful changes were observed in PD following the coaching change, the potential influence of player role (e.g., starters vs. substitutes) or rotation patterns under the different head coaches were not able to be analysed, but should be considered in future analyses encompassing different contextual factors. Fourth, only a single semi-professional male basketball team was recruited in this study. Therefore, further studies exploring players from professional levels and female competitions are warranted to understand the impacts of contextual factors on PD during matches given the unique physical outputs documented for these populations (Antoranz et al., 2025; Portes et al., 2020).

## Conclusions

These results suggest that, among the contextual factors explored, season phase was the most impactful on the PD experienced in matches among semi-professional, male basketball players. Specifically, the pre-season phase and promotion stage elicited higher PD across 30 s, 60 s, and 180 s time windows, likely due to the combination of tactical disorganization as teams prepared for the upcoming competitive season and the elevated opponent level, alongside the increased importance of matches that could determine promotion to a higher division. In contrast, match outcome and a mid-season change in head coach had negligible impacts on PD during matches, underscoring the importance of interpreting physical intensities within broader competitive and tactical contexts.

Although this study was exploratory in nature, the potential findings provide useful insights that practitioners may like to consider. Specifically, these findings highlight that contextualizing PD according to season phase may hold utility in load-based decision-making within elite basketball environments. In particular, preparing players for the potential elevated match demands observed during promotion phases may require exposure to comparable PD stimuli during training or preparatory match-play. Accordingly, practitioners should consider assessing the physical demands of training activities to ensure that load prescriptions appropriately reflect the specific requirements of each season phase. However, maximizing the physical intensities reached across brief periods may not solely translate to winning outcomes, due to the multi-factorial, complex nature of team performance in basketball. Conversely, the relatively high PD observed during pre-season should be interpreted cautiously, as these data may partly reflect contextual instability rather than stable competitive demands. Finally, a mid-season change to the head coach may not significantly impact the brief physical intensities reached in matches, but it is acknowledged that such changes may be made to alter other elements during play, such as team cohesion and tactical strategies.

## Data Availability

The data analyzed in this study is subject to the following licenses/restrictions: Due to ethical concerns with data restrictions further inquiries can be directed to the corresponding author. Requests to access these datasets should be directed to abel.inef.upm@gmail.com.
